# Supplementary material for: A happy accident: a novel turfgrass reference genome
Source: G3 (Bethesda). 2023 Apr 1;13(6):jkad073. doi: 10.1093/g3journal/jkad073 (PMC10234399; doi:10.1093/g3journal/jkad073)
Supplement: jkad073_Supplementary_Data [file jkad073_supplementary_data.zip › Supplementary_Figures_G3-2023-404165.pdf]

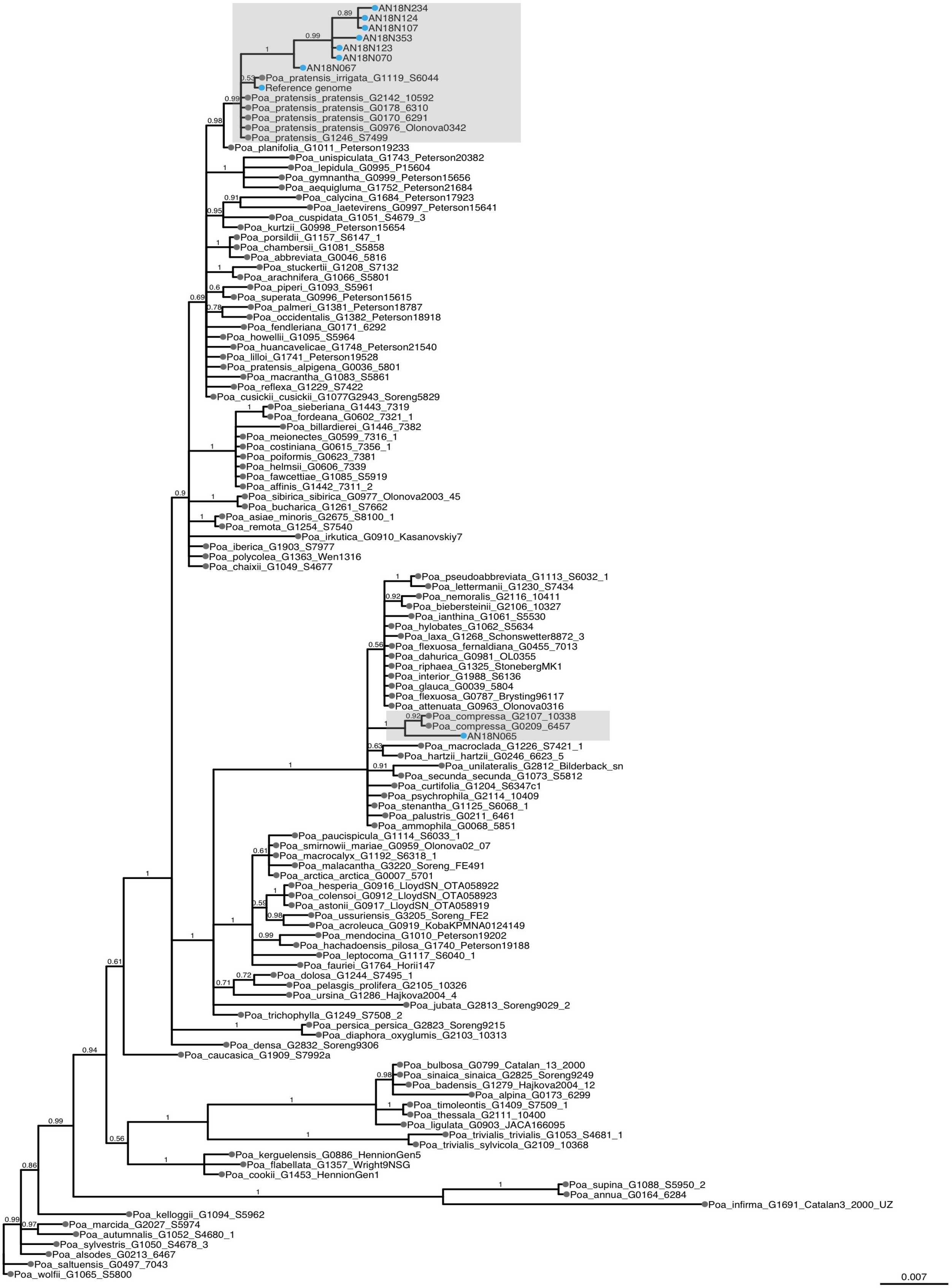

Figure S1 Bayesian 50% majority rule Consensus tree of ITS data. The *Poa* population panel and reference genome are indicated on the tree with blue dots. The unknown *Poa* population samples are labeled with their sample IDs (beginning with ’AN’). The shaded boxes indicate the two clades the reference genome and population panel group within: *P. pratensis* and *P. compressa*. Bayesian posterior probabilities shown above the branches and branch length is the expected substitutions per site.

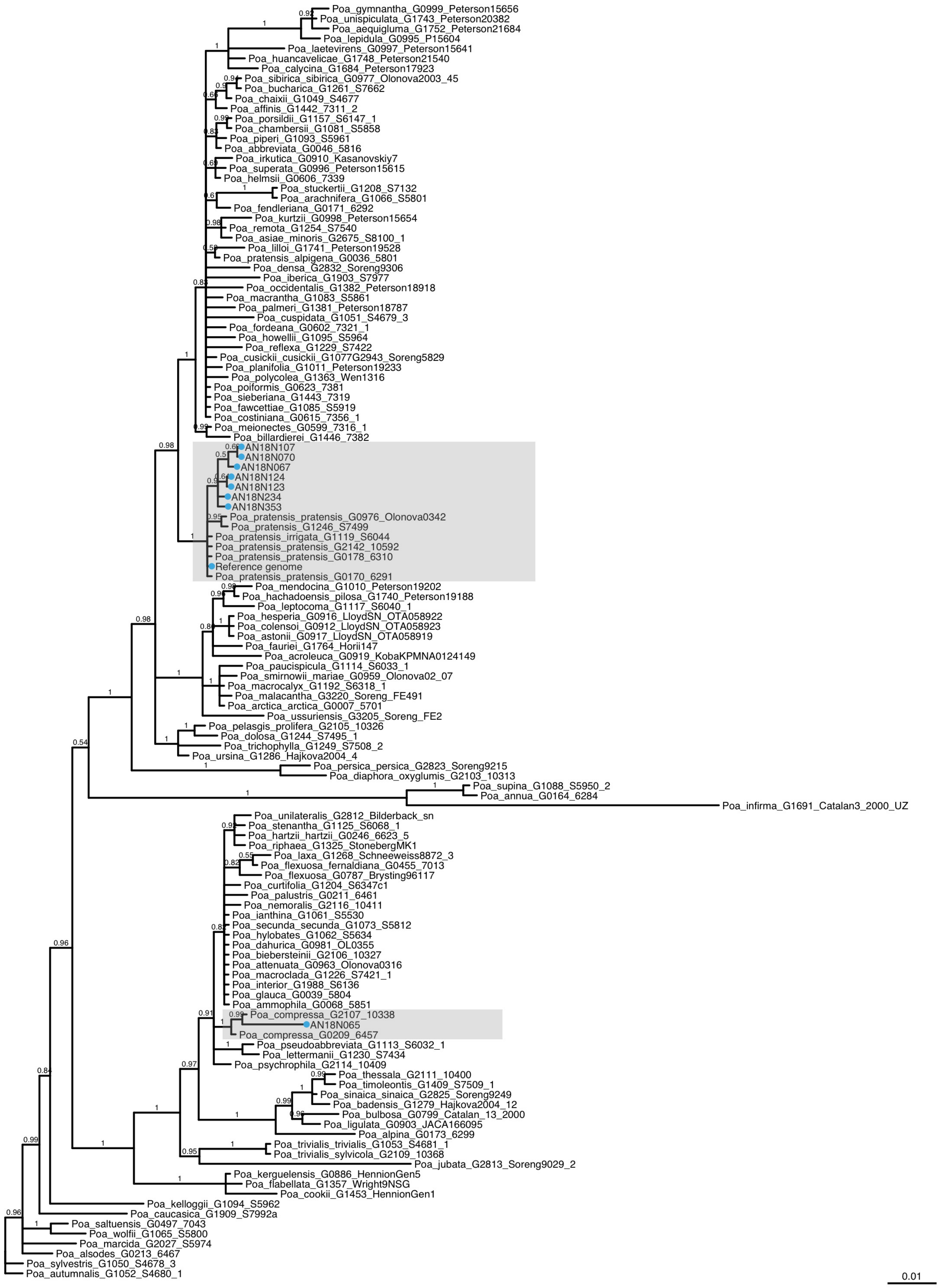

Figure S2 **Bayesian 50% majority rule consensus tree of ETS data.** See Figure S1 for description of the figure components.

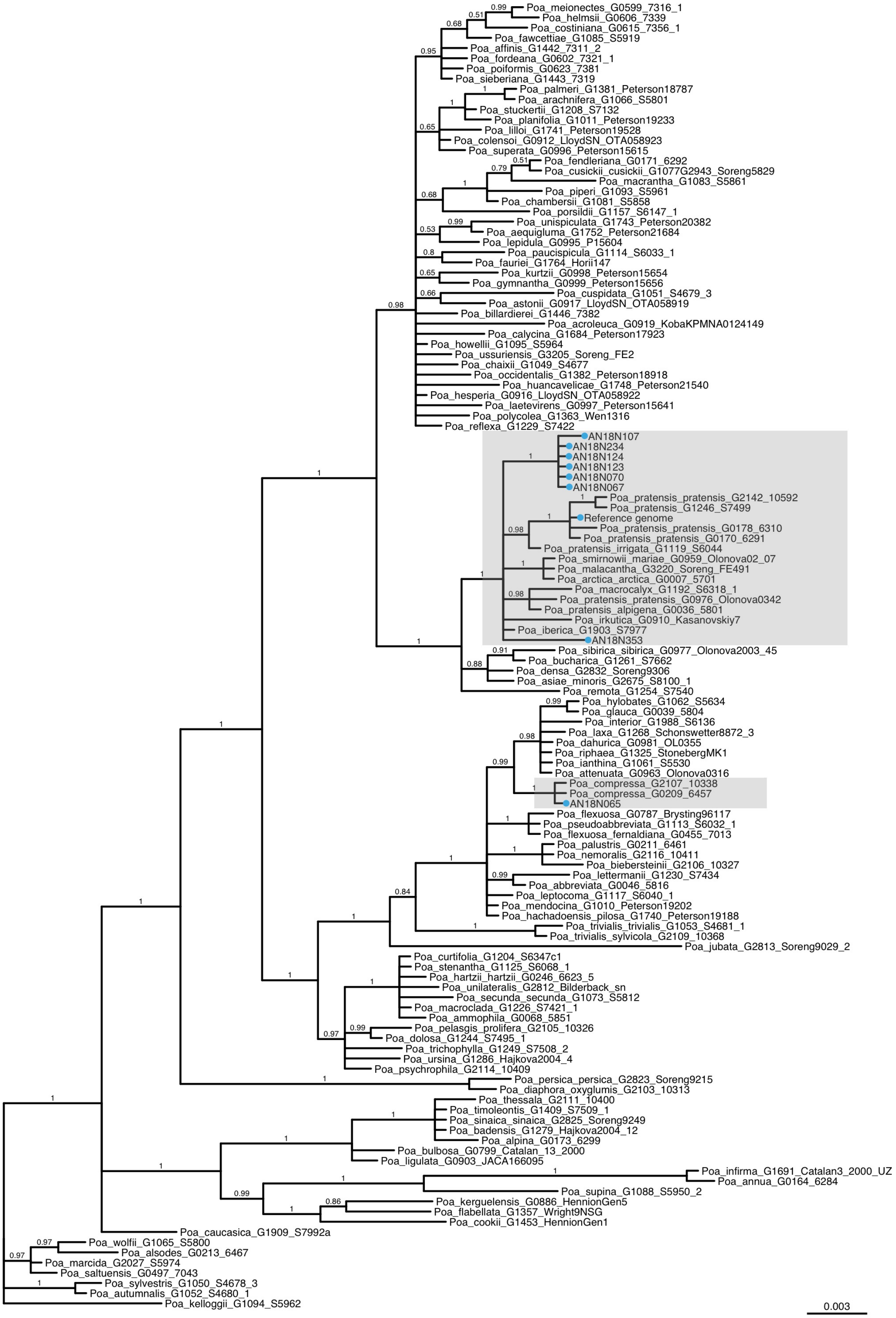

Figure S3 **Bayesian 50% majority rule Consensus tree of TLF data.** See Figure S1 for description of the figure components.

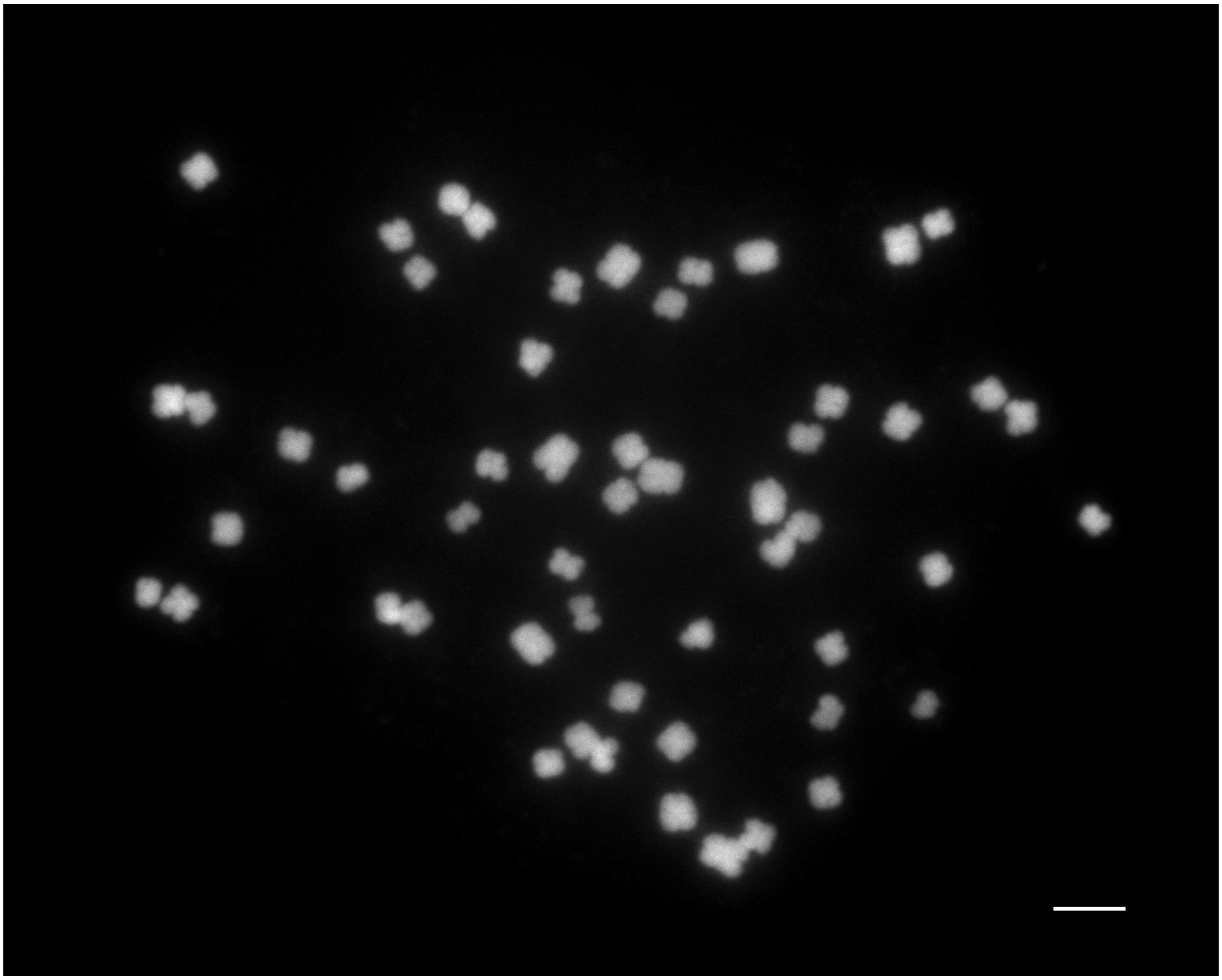

Figure S4 Metaphase chromosome spread with 54 chromosomes. *P. pratensis* reference individual root meristem cell counterstained with DAPI. Scale bar = 5 µm.

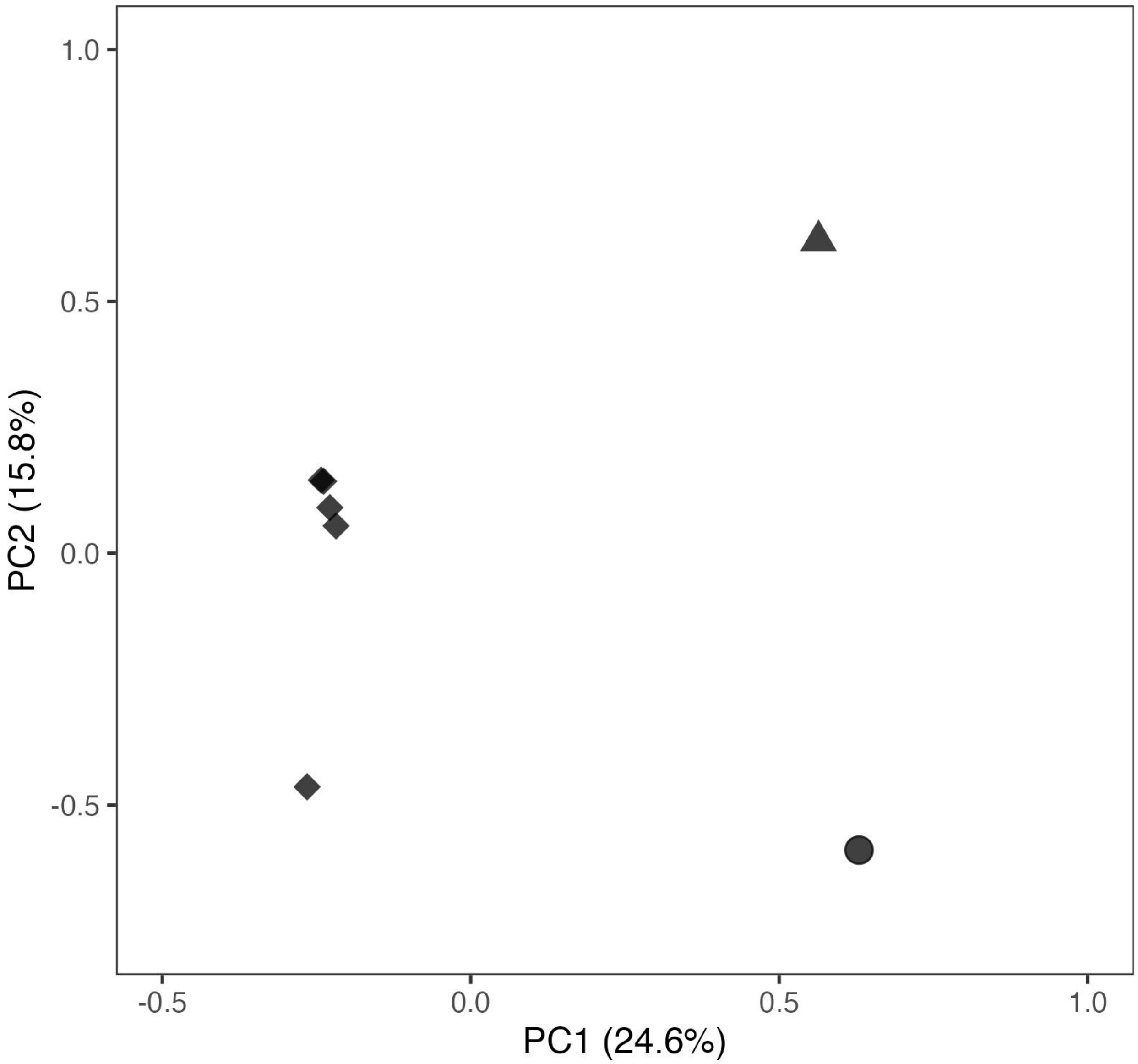

Figure S5 Population structure of *P. pratensis* genotypes only. The first two principal components (PCs) of a PCA of only the *P. pratensis* genotypes. The percent of genetic variation explained by each PC is reported in parenthesis on each axis. Sample locations are indicated by shape (circle = Argyle, Manitoba, triangle = Tolstoi, Manitoba, diamond = Boulder, Colorado).
